# Mechanistic insights into xanthine oxidoreductase from development studies of candidate drugs to treat hyperuricemia and gout

**DOI:** 10.1007/s00775-014-1210-x

**Published:** 2014-12-12

**Authors:** Takeshi Nishino, Ken Okamoto

**Affiliations:** 1 Department of Biochemistry and Molecular Biology, Nippon Medical School, 1-1-5 Sendagi, Bunkyou-ku, Tokyo, 113-8602 Japan; 2 Department of Applied Biological Chemistry, Graduate School of Agricultural and Life Sciences, University of Tokyo, 1-1-1 Yayoi, Bunkyo-Ku, Tokyo, 113-8657 Japan

**Keywords:** Xanthine oxidase, Uric acid, Allopurinol, Febuxostat, Gout

## Abstract

Xanthine oxidoreductase (XOR), which is widely distributed from humans to bacteria, has a key role in purine catabolism, catalyzing two steps of sequential hydroxylation from hypoxanthine to xanthine and from xanthine to urate at its molybdenum cofactor (Moco). Human XOR is considered to be a target of drugs not only for therapy of hyperuricemia and gout, but also potentially for a wide variety of other diseases. In this review, we focus on studies of XOR inhibitors and their implications for understanding the chemical nature and reaction mechanism of the Moco active site of XOR. We also discuss further experimental or clinical studies that would be helpful to clarify remaining issues.

## Introduction

**Fig. 1:**
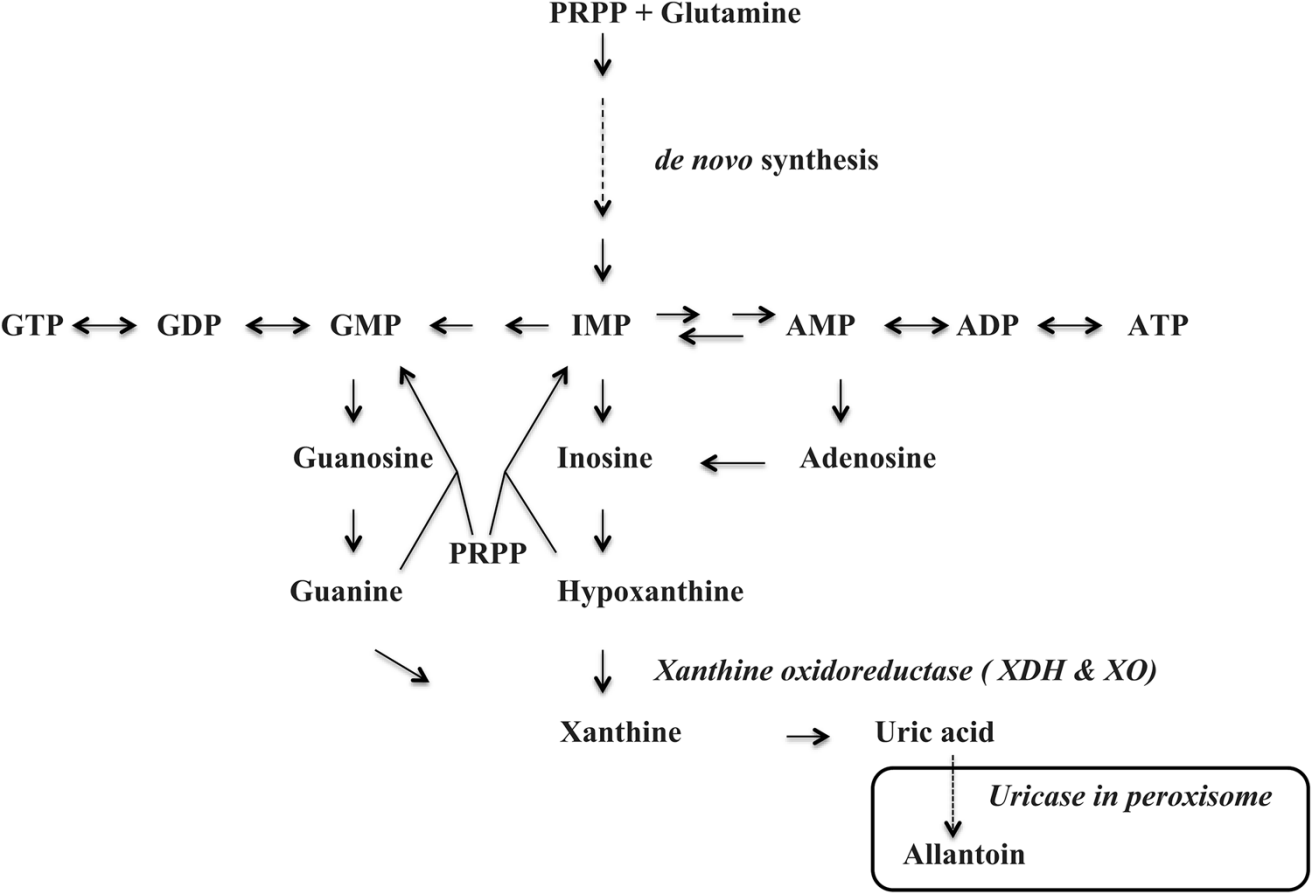
Schematic illustration of purine metabolism in primates. Hypoxanthine formed from inosine is hydroxylated to afford xanthine. Xanthine is also formed from guanine by deamination. Xanthine is further hydroxylated to uric acid (the final product in primates) in cytosol. Uric acid is converted to allantoin in peroxisomes of other mammalian species

Xanthine oxidoreductase (XOR), which can act as a xanthine oxidase or xanthine dehydrogenase, exists in a wide variety of organisms from bacteria to plants to humans [1, 2]. It catalyzes the reaction steps from hypoxanthine to xanthine and from xanthine to uric acid in the pathway of purine metabolism (Fig. 1); some aldehydes are also good substrates. The enzyme was first isolated from cow’s milk as aldehyde oxidase in 1902 [3], and was subsequently shown [4] to be the same as the enzyme previously identified as xanthine oxidase in milk [5]. XOR contains a molybdenum cofactor (Moco; molybdopterin), two non-identical [2Fe–2S] clusters, and one FAD per subunit of the dimeric protein, which has a total molecular weight (MW) of about 300 kDa. This enzyme is the best-studied complex flavoprotein, and several reviews are available [1, 2, 6–15].

In primates or uricoteric animals, uric acid is the end product of purine catabolism and is formed in cytosol [16]. However, in other organisms, e.g., in mammalians other than primates, uric acid is further oxidized to allantoin by uricase in peroxisomes [17–19]. Humans have lost uricase during evolution [20], and secrete uric acid mainly via kidney and partially via intestine. In kidney, uric acid is recovered via nephron transporter URAT1 [21] and also via ABCG2 transporter [22–24]. ABCG2 also mediates uric acid secretion from intestine, and a defect of this activity is one of the reasons for hyperuricemia [25]. A blood uric acid concentration higher than 7 mg/dL is a risk factor for gout [26] and is also proposed to be an independent risk factor for various diseases related to so-called metabolic syndrome [27], as well as kidney diseases [28] and endothelial dysfunction [29–32]. In order to decrease the concentration of uric acid in blood, drugs that inhibit biosynthesis or increase secretion of uric acid have been used clinically [33]. The XOR inhibitor allopurinol, an isomer of hypoxanthine, has been used to treat gout for more than 40 years, and the efficacy of XOR inhibition as a strategy for prevention of gout attack is well established. However, in some cases allopurinol causes serious adverse effects [34, 35], and so new inhibitors of uric acid formation have been sought to replace it in such cases. Indeed, a patent survey indicated that more than 250 XOR inhibitors have been investigated as candidate drugs [36]. However, none of them, other than allopurinol, has been approved until quite recently: febuxostat was approved in EU countries in 2008, in the USA in 2009, and subsequently in various other countries including Japan (2011). Another inhibitor, topiroxostat, has also been approved recently in Japan (2013). In this paper, we review the implications of enzymological and developmental studies of XOR inhibitors for understanding the chemical nature and reaction mechanism of the Moco active site of XOR.

## Brief overview of XOR

**Fig. 2:**
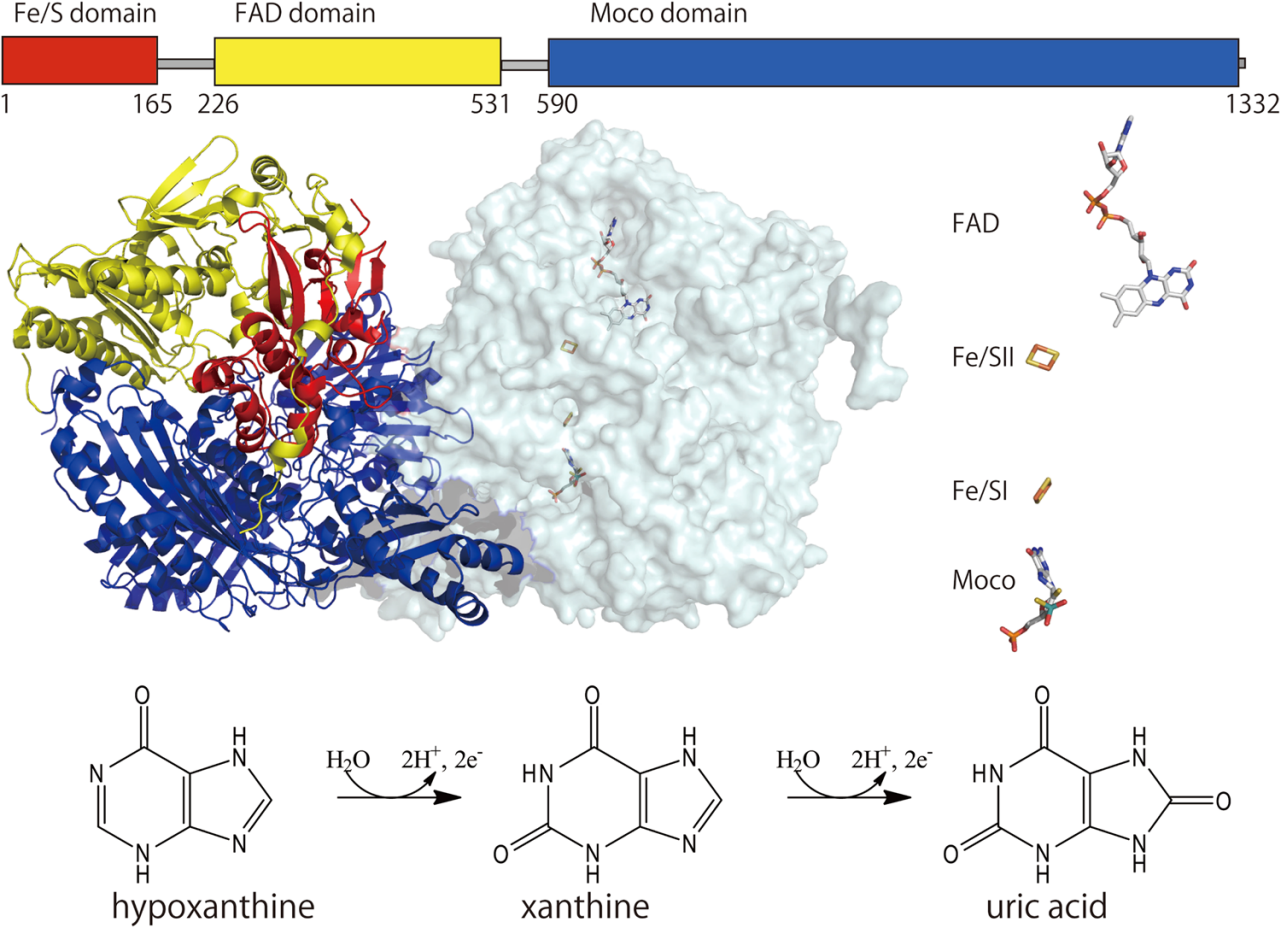
Structure of bovine XOR. *Top* Primary structure of bovine XOR subunit illustrated as three domains connected with two linker peptides. The N-terminal (*red*), the C-terminal (*blue*) and the intermediate (*yellow*) domains contain the iron–sulfur centers, the Moco center and the FAD center, respectively. *Middle left* Homodimer structure of bovine XOR illustrated with one subunit as a ribbon model and the other as a space-filling model. *Right* Cofactor arrangement. Figures were generated from PDB ID 1F4Q. *Bottom* Hydroxylation reactions of hypoxanthine to xanthine and xanthine to uric acid. Two electrons are transferred to the Mo atom of Moco

Mammalian XOR exists as a homodimer of 150 kDa subunits; its three-dimensional and primary structures are illustrated in Fig. 2. Each of the subunits is composed of three domains. The largest, C-terminal domain contains the molybdenum cofactor (Moco), the intermediate domain contains flavin adenine dinucleotide (FAD) cofactor and the smallest, N-terminal domain contains the two iron sulfur clusters ([2Fe–2S] type). The redox reaction centers are positioned in the order of Moco, the two iron clusters and FAD with an overall separation of less than 14 Å, as is usually the case for electron transfer components [37–41]. The iron sulfur clusters are designated Fe/S I and Fe/S II [42–45]; Fe/S II has the higher redox potential, and shows unusually broad EPR signals at temperatures below 22 K, whereas Fe/S I is typical plant ferredoxin-type center [44]. Assignment of these clusters in the primary structure was performed by means of site-directed mutagenesis studies [43]. The hydroxylation reaction of purine takes place at Moco; firstly, two electrons are transferred from the substrate to Mo of Moco, reducing Mo(VI) to Mo(IV); Mo(V) is observed only transiently. It is considered that H^+^ + 2e^−^ derived from the substrate, in the form of hydride (H^−^, i.e., a hydrogen atom with two electrons) is transferred to a sulfur ligand of Moco (Mo(VI) = S → Mo(IV)–SH) [1, 46, 47]. The Mo(V) species is not formed by one-electron transfer from the substrate, but rather is formed during the process of electron transfer from Mo to the iron sulfur center. Electrons are transferred to FAD via the iron sulfur centers in the order illustrated in Fig. 2. Finally, NAD^+^ or oxygen molecule receives an electron from FAD to afford NADH or H_2_O_2_ and O_2_^−^, respectively. XOR from most organisms uses NAD^+^ as an electron acceptor and is called xanthine dehydrogenase (XDH), while XOR proteins from mammals can be converted to the oxidase form (XO), which uses exclusively the oxygen molecule as an electron acceptor [48–52]. The conversion involves posttranslational modification such as disulfide formation or proteolytic nicking of the same XOR gene product [2, 53–55]. Historically, XOR was isolated from cow’s milk after irreversible conversion to XO by nicking the enzyme with pancreatin to isolate it from milk fat globule membrane [56], so the enzyme has been called ‘xanthine oxidase’, but it is considered that the enzyme actually exists in xanthine dehydrogenase form [48, 57]. The physiological and pathological roles of the conversion have long attracted attention, particularly from researchers in the medical field, but remain controversial [6, 12, 14, 58], although the mechanism of the conversion is well understood and has been reviewed in detail [10].

## Inhibitor characteristics and inhibitory mechanism

### Allopurinol

**Fig. 3:**
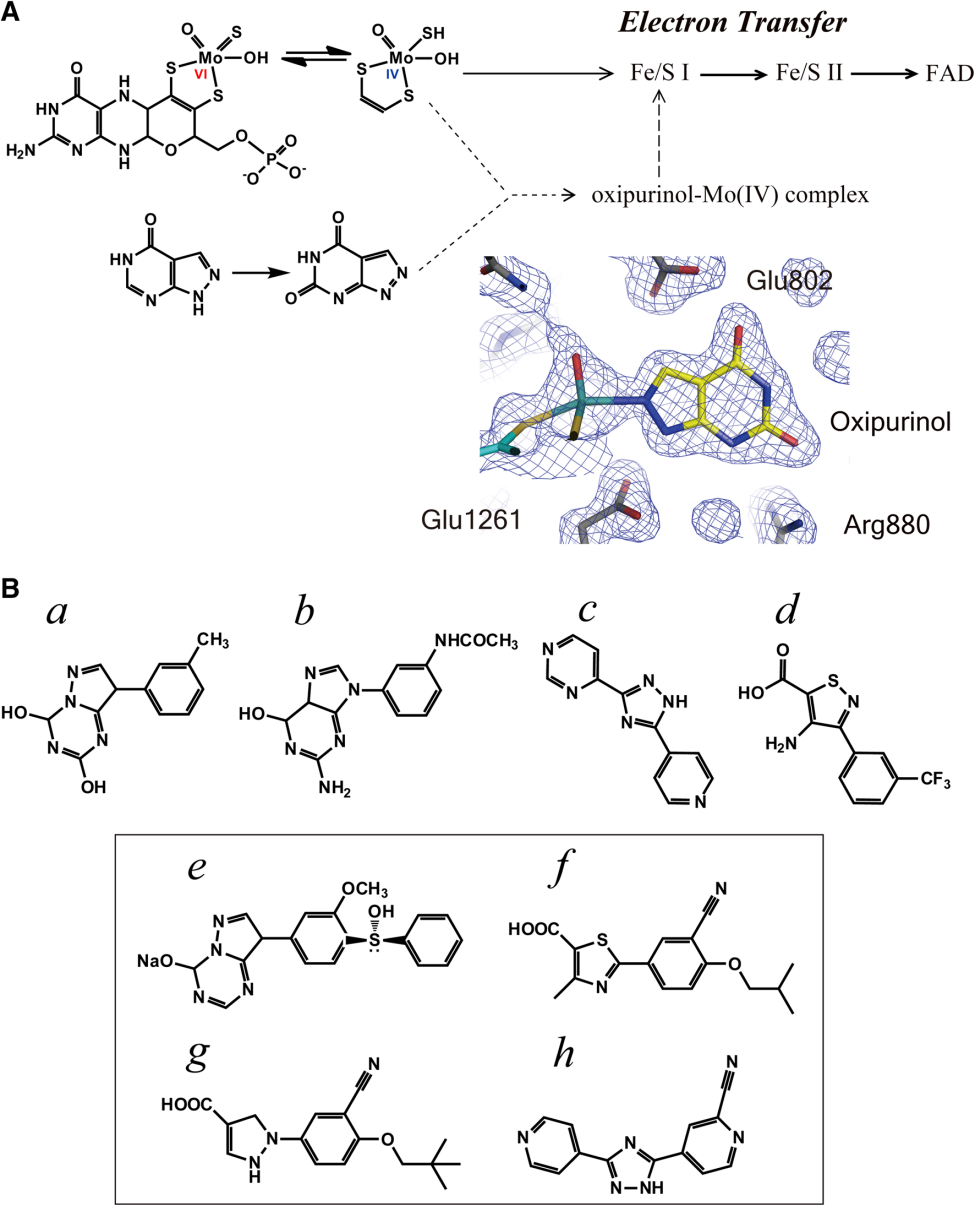
Inhibitors of XOR. **A** Mechanism of inhibition of XOR by allopurinol. Allopurinol is a good substrate of XOR and is converted to oxipurinol with concomitant reduction of Mo (VI) to Mo(IV). Mo(IV) is mainly re-oxidized via electron transfer to the other cofactors in XOR. During turnover, Mo(IV) reacts with oxipurinol to form a tightly bound complex; its crystal structure is shown as an electron-density map. **B** Representative potent inhibitors reported after the clinical application of allopurinol. (*a*) from ICN Pharma: IC_50_, 25 nM; (*b*) from C. Silipo: IC_50_, 10 nM; (*c*) from E. Merck: IC_50_, 40 nM; (*d*) from Eli Lilly: IC_50_, 13 nM. In *box* inhibitors that have been examined in detail, including crystal structure of the XOR-bound form. (*e*) BOF-4272; (*f*) febuxostat, TEI-6720; (*g*) pyranoxostat, Y-700; (*h*) topiroxostat, FYX-051

Allopurinol (4-OH-pyrazolo-pyrimidine) has been used as an anti-gout drug for over 40 years. It was synthesized by Robins [59] and introduced into clinical use by Elion et al. [60]. It is an isomer of hypoxanthine, and was initially reported to be a simple competitive inhibitor that binds to the molybdenum center competitively with respect to xanthine, with the *K*_*i*_ value of 7 × 10^−7 ^M for the rat enzyme and 1.9 × 10^−7 ^M for the human enzyme [61]. The IC_50_ value was reported as 1,700 nM [36]. However, it subsequently became clear that the inhibitory mechanism of allopurinol is more complicated and potent than initially envisaged [62, 63]. Massey et al. [63] showed that the inhibition progresses in a time-dependent manner, with eventual formation of a tightly bound complex of the reduced enzyme (MoIV) with oxipurinol (often called alloxanthine) generated by hydroxylation of allopurinol, as illustrated in Fig. 3A. The reason for the time dependence of the inhibition is the time taken to convert allopurinol to oxipurinol and to trap reduced MoIV that is transiently formed during enzymatic turnover. The oxipurinol–molybdenum complex dissociates upon re-oxidation of Mo(IV) in air (*t*_1/2_ = 300 min at 25 °C) due to electron transfer to oxidized cofactors [63, 64]. Massey et al. [63] also concluded that the naturally isolated enzyme contained a significant amount of inactive form that could not bind oxipurinol, since the molar ratio of oxipurinol per enzyme FAD was less than one, and was correlated linearly to the specific activity of various enzyme samples. They found subsequently that the inactive form is identical to the inactive enzyme generated by treatment with KCN, which releases a sulfur atom at the active site that is essential for catalytic activity. They also found that the inactive enzyme could be reactivated to the active sulfo form by Na_2_^35^S treatment, with incorporation of a stoichiometric amount of ^35^S into the enzyme [65, 66]. Thus, studies on the XOR inhibitor allopurinol yielded the important finding that the sulfide ligand is essential for enzymatic activity. It was subsequently shown that sulfide is one of the coordinating ligands of the Mo atom [67]. Separation of the active and inactive forms of the enzyme was attempted by Edmondson et al. [68] using an affinity-gel-linked allopurinol analog with a long-chain ligand at the 5-position. On the other hand, Nishino et al. [69, 70] used allopurinol in a rather different way: they applied the Mo(IV)–oxipurinol enzyme complex to folate affinity gel, which binds the free active site of any enzyme species, including the inactive desulfo form, and then they reactivated the passed-through fraction of Mo(IV)–oxipurinol complex by oxidation with ferricyanide. Thus, studies using allopurinol provided important information about the presence of the inactive desulfo form in the naturally isolated enzyme and the action mechanism of allopurinol as a suicide inhibitor, as well as providing a basis for separation of inactive enzyme. Before discovery of the desulfo form, Bray et al. had reported that XO isolated from bovine milk contains an inactive demolybdo form in a small amount that depended on the season and the location where the cows had grazed. These results suggested that the content of demolybdo form depends on the nutritional status of cows [71]. Nishino et al. [69] also noted that XOR isolated from Japanese cows’ milk contains a very small amount of inactive demolybdo form that can be isolated by first folate affinity column chromatography (passed-through fractions), as shown previously. Similar results were observed for American cows’ milk (unpublished observations). On the other hand, Harrison et al. [72, 73] reported that XOR isolated from human milk was mostly in demolybdo form. In contrast, however, Moriwaki et al. [74] or Krenitsky et al. [75] reported purification and characterization of human liver enzyme and showed that its activity is not greatly different from that of normal bovine milk XOR. The content of inactive enzyme is an important issue in evaluating the effects of XOR inhibitors in vivo, since structure-based inhibitors can bind to inactive enzyme species, as described later, and this would decrease the efficacy of drugs by lowering the free inhibitor concentration in blood.

### Inhibitors other than allopurinol

Various inhibitors were reported after the introduction of allopurinol, but most of them have not been approved for clinical use. Potent inhibitors identified from a patent survey or reported in the literature [76–79] are shown in Fig. 3B, together with their IC_50_ values (inhibitor concentration required to inhibit the enzyme activity by 50 %). Other new XOR inhibitors have also been reported in the last few years (structures not shown here) [80–92]. However, it should be pointed out that the IC_50_ values are not necessarily reliable. For example, as described in the previous section, allopurinol is a potent suicide inhibitor, so its potency cannot be shown in terms of IC_50_, i.e., inhibition is dependent upon incubation time, enzyme concentration and percent of active form of the enzyme. In most cases, detailed enzymological or pharmacological data for the inhibitors were not reported, except in the case of 2-phenyl-4-methyl-1,3-selenazole-5-carboxylic acid (PMSC), for which *K*_*i*_ and *K*_*i*_′ values were given, in addition to a docking study [89]. Here, in order to illustrate the utility of XOR inhibitor studies for understanding the chemical nature and reaction mechanism of the enzyme, we will focus on the inhibitors BOF-4272, TEI-6720, Y-700 and FYX-051, which were characterized in detail using fully active bovine XO after the mid-1980s (Fig. 3B in box). The last compound will be described in the next section.

#### BOF-4272

Sodium-8-(3-methoxy-4-phenylsulfinylphenyl)pyrazolo[1,5-a]-1,3,5-triazine-4-olate monohydrate (BOF-4272) is separable into the (−)-isomer and (+) isomer by HPLC using a Chiralcel OD column. The (−)-isomer showed much higher affinity for the enzyme, and steady-state kinetics showed mixed-type inhibition with *K*_*i*_ = 1.2 × 10^−9^ M and *K*_*i*_′ = 9 × 10^−9^ M for xanthine and oxygen as substrates, whereas the (+)-isomer showed *K*_*i*_ = 3 × 10^−7^ M and *K*_*i*_′ = 9 × 10^−6^ M [93]. A plausible explanation for the mixed-type inhibition is the difference of *K*_*i*_ for oxidized and *K*_*i*_′ for reduced enzyme [93], and this may also be the case for other inhibitors (Fig. 3B in box). Although BOF-4272 was a potent inhibitor in animal experiments [94], clinical studies indicated that its efficacy varies from individual to individual; in some cases it was as effective as allopurinol on a weight basis, but in others it was less effective. It was concluded that the drug concentration in blood was insufficient, probably due to hepatic metabolism, as well as poor absorption of the drug from the intestine. Overall, this work indicated that uric acid production in humans occurs not only in liver, but also to a significant extent in other organs, whereas other animals produce uric acid predominantly in liver. Thus, it may be necessary to use a more potent inhibitor (*K*_*i*_ value less than 10^−9^) and to achieve a higher concentration of inhibitor in blood to obtain clinical efficacy. The lessons learned during the work on BOF-4272 contributed greatly to the development of the following inhibitors as clinically useful drugs.

#### *Febuxostat* (TEI-6720; (2-[3-cyano-4-isobutoxyphenyl]-4-methyl-5-thiazolecarboxylic acid) [105] and *pyranostat* (Y-700; 1-[3-cyano-4-(2,2-dimethylpropoxy)phenyl]-1H-pyrazole-4-carboxylic acid)

These compounds were synthesized and selected by Kondo et al. [95] and Fukunari et al. [96] at Teijin Co. and Mitsubishi Pharma Co., respectively, from among various synthesized compounds based on the criteria of *K*_*i*_ value less than 10^−9^ M using fully active enzyme and good solubility. Both of them showed mixed-type inhibition in steady-state kinetic studies using fully active enzyme, like BOF-4272. It should be noted that inhibition of XOR by febuxostat is not linear with time [95], so steady-state analysis based on initial velocity was employed. The *K*_d_ value of XOR-febuxostat was too low to measure, even with a sensitive florescence detection method. Based on the crystal structure and MD simulation, it was proposed that the protein structure around the Moco cavity has a dynamic open structure in solution, but adopts a more compact structure after binding of the inhibitor in the protein cavity [97]. This idea is consistent with the fact that we have succeeded in crystallizing many mammalian XOR proteins, including mutants, only in the presence of additional compounds such as inhibitors. XOR in which the Moco cavity is unoccupied has never been crystallized.

## Crystal structures of XOR bound with salicylate, oxipurinol, BOF-4272, febuxostat, and pyranostat

### Salicylate

**Fig. 4:**
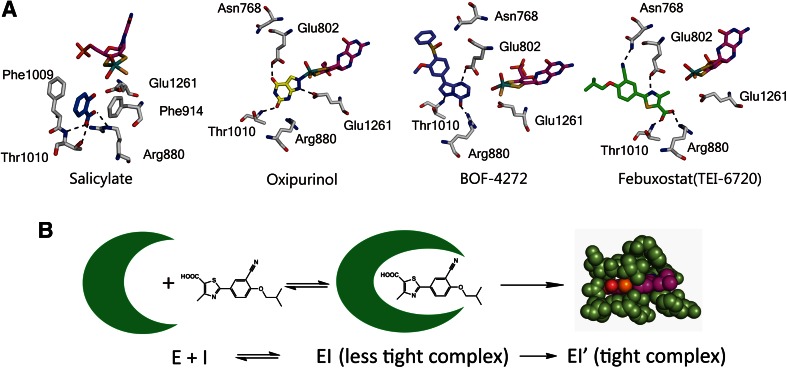
Crystal structures of potent inhibitors bound to the XOR Moco center. **A** Inhibitors and amino acid residues are illustrated by stick models with atom colors reproduced from PDB. Salicylate, PDB:1FO4; oxipurinol, PDB:3BDJ; BOF-4272: data deposition in process; Febuxostat, PDB:1N5X. **B** Schematic model of interaction of febuxostat with the active site cavity. Interaction of febustostat with the open cavity affords a weakly bound complex with the *K*_*i*_ values determined by steady-state kinetics using initial velocity, and this is subsequently converted to a tightly bound complex, of which the *K*_d_ value is too low to be determined accurately (see text)

The crystal structure of bovine XOR was first determined as the salicylate-bound form (Fig. 4A) [37]. Salicylate, a weak competitive inhibitor, is normally included during purification to prevent conversion of sulfo-XOR to inactive desulfo-XOR. In the XDH crystal structure, the salicylate molecule is bound 6.5 Å from the Mo ion, in a position that would be overlapped by larger aromatic substrates (hypoxanthine or xanthine) present at the binding site. Thus, although salicylate itself does not bind to the Moco, it blocks the approach of substrates to the metal complex. Salicylate binding is mediated by several hydrogen bonds and electrostatic interactions. The inhibitor is aligned parallel to the phenyl ring of Phe 914, but the two aromatic rings only slightly overlap. At the same time, the phenyl ring of Phe 1009 interacts edge-on with the center of the salicylate ring. Both of the carboxylate atoms are close to the guanidinium group of Arg 880 and they also interact with the hydroxyl side chain of Thr 1010 and via a water molecule with the carboxylate of Glu 1261. The salicylate hydroxyl group forms hydrogen bonds to both the backbone amide and hydroxyl side chain of Thr 1010.

### Oxipurinol (Allopurinol)

The crystal structure of the complex of pre-reduced fully active bovine XO with oxipurinol was determined at 2.0 Å resolution [98]. Strong electron density connected oxipurinol and the Mo atom (Fig. 3A). The N_2_ nitrogen of oxipurinol replaces the equatorial hydroxyl ligand of the molybdenum atom, coordinating directly to the metal atom at a distance of 2.3 Å with a 180° angle between Mo-ion, N_2_-atom and the pyrazole ring (Figs. 3A, 4A). The other position is occupied by the –SH atom located at a distance of about 2.4 Å from the central metal ion, in accordance with the previously described mechanism of inhibition by allopurinol. Oxipurinol forms hydrogen bonds with Glu802 via its 4-position (=O; corresponding to the 6-position of xanthine), Arg880 via its 6-position (=O; corresponding to the 2-position of xanthine) and Glu1261 via N1. These interactions provide clues to the binding mode of substrate xanthine, an isomer of oxipurinol, as will be discussed below.

### BOF-4272, Febuxostat and pyranostat

The crystal structures of the complexes of milk XOR with BOF-4272, febuxostat and pyranostat show features that account for the tight binding; these inhibitors fit well into the substrate-binding channel of XOR, and no open space remains in the channel after binding of these compounds. Docking study of PMSC, which has higher potency than febuxostat, probably due to replacement of S by Se, suggested that PMSC may also fill the space in the same region, although the crystal structure was not available [80]. In contrast to oxipurinol, febuxostat and pyranostat do not form a covalent bond with molybdenum. However, multiple weak interactions, such as ionic and hydrogen bonds, *π*–*π* interactions between the main five-membered ring and nearby phenylalanine residues, van der Waals interactions, and hydrophobic interactions [95], result in tight binding; the dissociation constants are very low. Moreover, the fit of these compounds to the enzyme’s active-site structure is enhanced by rotation of the region between the five-membered ring and the benzene ring. Thus, these inhibitors efficiently match the structure of the substrate-binding region of the enzyme. A hydrogen-bonding interaction of the CN group of the inhibitors with an asparagine residue of the enzyme should be noted. In the crystal structure, the side chain amide of Asn768 and the CN group at the 3-position are only ~3 Å apart [94, 96]. Although this asparagine residue is located too far from the active center for direct involvement in purine substrate recognition or catalytic activity, the CN group of these inhibitors is necessary for potent enzyme inhibitory activity. A bulky hydrophobic moiety at the 4-position is also essential for tight binding. The 4-isobutoxy group is surrounded by hydrophobic amino acids at distances of around 4 Å [96]. Interestingly, these crystallographically determined features of the inhibitor binding mode suggest that the fit of the inhibitors in the cavity is too tight to allow entry of the inhibitors into the cavity, as shown in Fig. 4B, suggesting that initially the inhibitors bind rather weakly to an open form of the dynamic protein structure. The *K*_*i*_ and *K*_*i*_′ values determined by steady-state kinetics using initial rate would reflect this, while the *K*_d_ values for the subsequent tight interaction are too low to determine accurately. Interestingly, these structure-based inhibitors do not efficiently inhibit bacterial XOR, even though its crystal structure is very similar and important amino acid residues involved in catalysis are conserved. MD simulation study indicated that the mobility of the active-site cavity is altered due to replacement of amino acid residues not involved in catalysis, providing further evidence for the dynamic flexibility of Moco in the active site [97].

### Topiroxostat: (FYX-051, 4-[5-Pyridin-4-yl-1H-[1, 2, 4] triazol-3-yl]pyridine-2-carbonitrile)

**Fig. 5:**
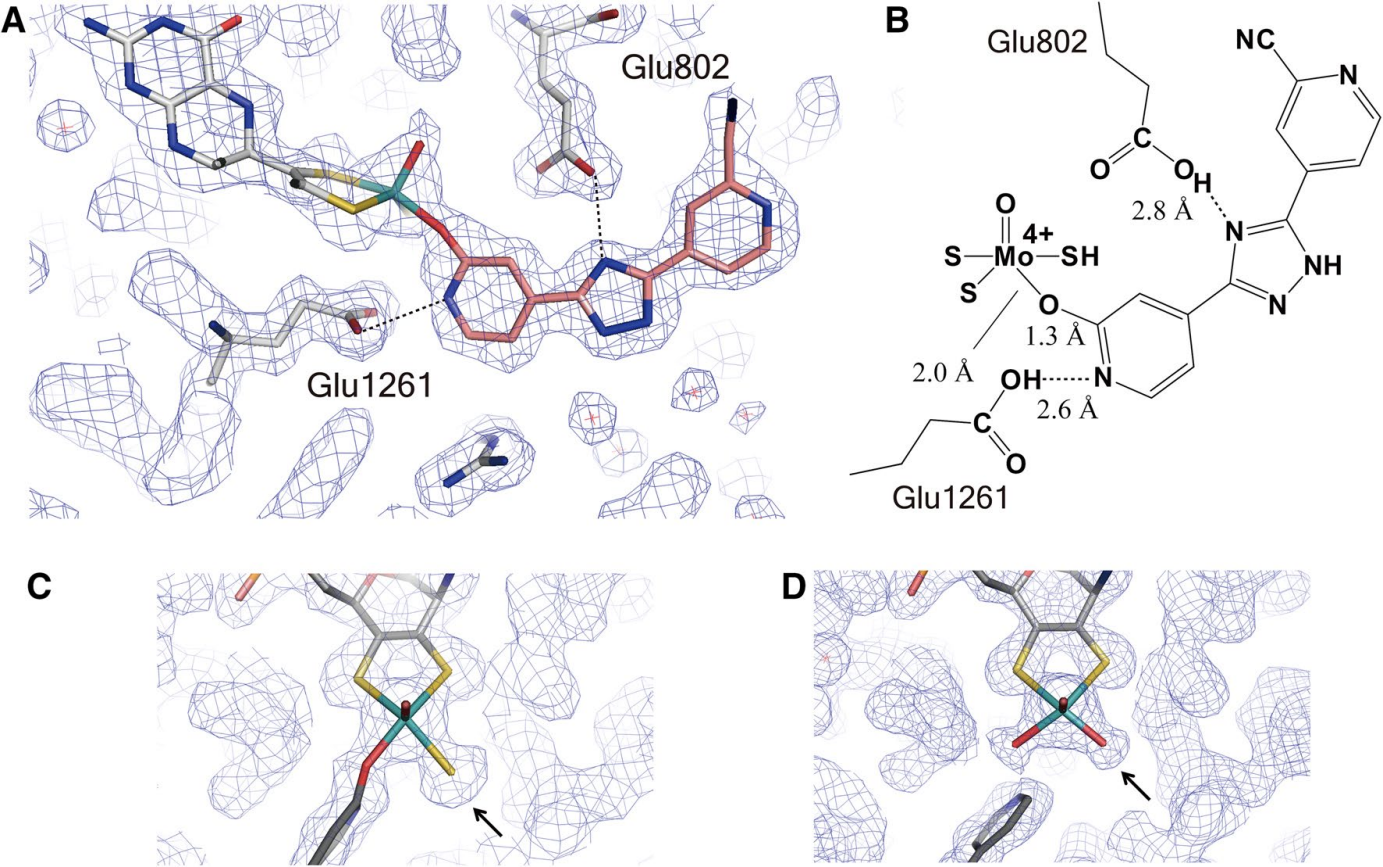
Crystal structure of topiroxostat (FYX-051) bound to XOR and binding modes of substrates. **A** Electron-density map around the Mo atom in the complex of fully active XOR with topiroxostat. **B** Interaction of amino acid residues in the protein cavity. Two protonated glutamate residues, E802 and E1261, are at hydrogen-bonding distances from N atoms of topiroxostat. **C** Electron density around the Mo atom in the complex of fully active XOR with topiroxostat, showing electron density between Mo and a carbon atom of topiroxostat. See similar electron densities of two sulfur atoms connected to pterin moiety. **D** Electron density around the Mo atom in the case of inactive desulfo-XOR, showing no electron density between Mo and a carbon atom. Lower electron density is observed, similar to that another oxygen atom coordinated to the equatorial position. Both are viewed from the apical position. Differences in electron density are indicated by *arrows*

It is a potent inhibitor that serves as a suicide substrate of XOR [99, 100] and also has similar features to structure-based inhibitors such as febuxostat and pyranostat. Topiroxostat transfers two electrons to the enzyme, forming a stable complex with Mo(IV) within a short time [98]. In the crystal structure of the complex of fully active XDH and topiroxostat at 1.9 Å resolution (Fig. 5A, B), it was seen that one of the C atoms of topiroxostat, next to the N atom, forms a covalent bond with molybdenum through oxygen. The bridging electron density between the Mo and C atoms is bent at an angle of 152°, and spans 3.3 Å in total (Mo–O, 2.0 Å and O–C, 1.3 Å), indicating Mo–O–C bond formation. It had not been possible to identify definitively the chemical nature of atoms bound to the Mo atom based on electron density alone in the previous lower-resolution crystal structure with XDH that was not fully active [37], but three ligands of the Mo atom were tentatively assigned as =S, =O, and –OH according to the previously proposed geometry [101]. However, at higher resolution, with fully active XDH, the topiroxostat-inhibited XDH structure provided an updated geometry of the Mo ligands and the ligands were definitely assigned as =S (–SH in reduced Mo) and –O–C– at the equatorial position, and =O at the apical position from reduced Mo (IV), as shown in Fig. 5, by comparison with the structure of desulfo-XO bound with the same inhibitor (Fig. 5C, D). This updated geometry is consistent with magnetic circular dichroism findings [102], X-ray absorption spectroscopy [103] and analysis of model compounds. Various interactions, including hydrogen bonding with surrounding amino acids, hydrophobic interaction and *π*–*π* stacking interaction with two phenylalanines, were observed, similarly to the cases of febuxostat and pyranostat. Mo(IV)–O–C– of the initially formed complex of Mo(IV)–topiroxostat decomposed with a half-life of approximately 20 h at 25 °C and formed another complex interacting in different manner [100]. The structure of this complex provided important insight into the hydroxylation mechanism. An identical structure was formed when dithionite-reduced Mo(IV) was mixed with hydroxy topiroxostat. It was shown that protonated Glu1261 forms a hydrogen bond with substrate nitrogen next to the carbon atom to be hydroxylated, suggesting an important role of protonated Glu1261, i.e., hydrogen bonding to the nitrogen atom facilitates nucleophilic attack on adjacent carbon by the basic oxygen atom (Mo–O–) [99]. This concept is consistent with the role of the side chain of a glutamic acid residue near the Mo–OH group in the reaction of aldehyde oxidoreductase (ALO) from the sulfate-reducing anaerobic bacterium *Desulfovibrio gigas* [101]. Thus, it can be expected that a C atom of various *N*-heterocyclic compounds can be hydroxylated, if the compounds have affinity for the active-site cavity. We believe that physiological substrates are also activated in essentially the same manner. When this glutamic acid residue was altered by mutagenesis to alanine, the activity was completely lost [104]. The rate-limiting step is cleavage of the Mo–O–C bond by a water molecule to give Mo–OH plus C–OH, but no such water molecule was observed in the crystal structure of the topiroxostat–XOR complex, probably because the site is fully occupied by a bulky hydrophobic molecule [99].

## Mechanism of hydroxylation of purine substrates

**Fig. 6:**
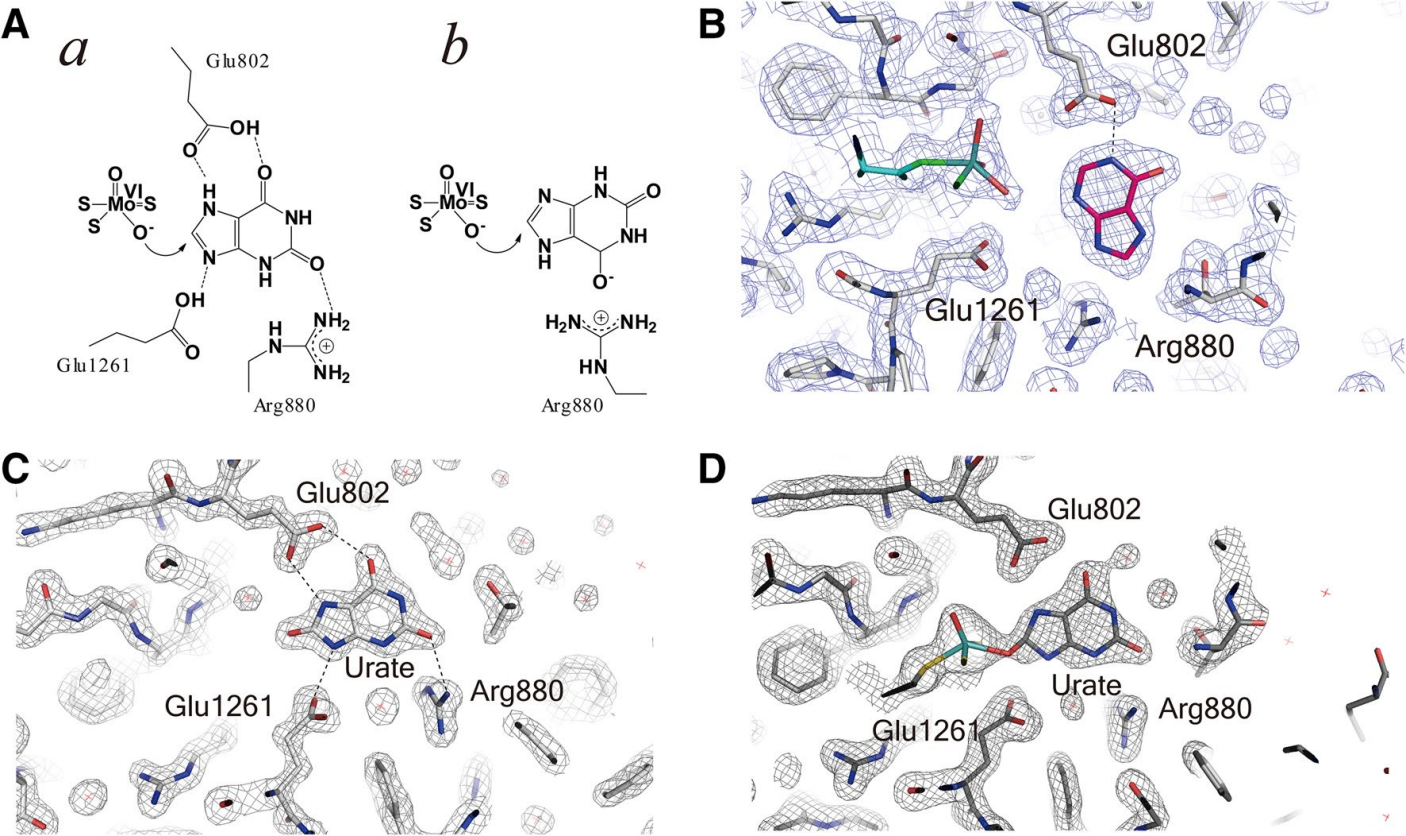
Crystal structure of XOR with oxipurines. **A** Two binding models have been proposed. (*a*) Binding mode proposed by the authors of this paper. (*b*) Binding mode proposed by Hille et al. and Kisker et al. **B** Binding mode for the complex of desulfo-XOR with hypoxanthine obtained by the authors of this paper. **C** Crystal structures urate bound to demolybdo-XOR. **D** Urate bound to reduced Mo of fully active XOR

We previously reviewed the hydroxylation mechanism at the Moco site in this journal in 2008 [10]. As described then, two contradictory models have been proposed for the substrate-binding mode and activation mechanism. We revisit this issue here because, despite further research [105–108], the issue remains controversial [15, 106, 109]. The first model shown in Fig. 6A-a is based on site-directed studies [40], the binding mode of oxipurinol explored by X-ray structure analyses with *R.* *capsulatus* XDH [38] and with bovine XOR [98], and the mechanism in the case of topiroxostat [99]. Nucleophilic reaction is activated or facilitated through hydrogen bonds formed between the substrate and amino acid residues, particularly by Glu1261, as described in the section on topiroxostat. The same authors subsequently presented X-ray data at high resolution showing the binding mode of demolybdo-XOR and urate [105]. These binding modes are consistent with the metabolic sequence, i.e., hydroxylation at the 2-position of hypoxanthine precedes that at the 8-position. That is, interaction of the 2-position keto group (C=O) and Arg881 is important for efficient hydroxylation at the 8-position, as discussed in connection with mutagenesis studies [10, 40]. The other mode does not have such an interaction. This is consistent with the report that mutation of the corresponding arginine to glutamate in *A. nidulans* XOR resulted in loss of xanthine hydroxylation activity, though activity towards hypoxanthine still remained [110].

Another binding mode and activation mechanism were proposed by Hille et al. [111, 112] (Figure 6A-b), in which negative charge can be accumulated at the 6-position oxygen through interaction with the positive charge of the guanidinium group of Arg880; this would be energetically more favorable. A classification scheme for bad (slow) (Fig. 6A-a) and good (fast) (Fig. 6A-b) substrates was proposed based on their orientation in the active site, in the light of experimental data for 2-hydroxy-6-methylpurine as a substrate and for the xanthine-bound desulfo form of bovine XO. The former structure was considered to be a poor orientation, and therefore the reaction is slow due to high activation energy. However, the slower rate is not due to a slower rate of hydroxylation, but rather is due to a slower rate of release of the product [113].

Theoretical calculations using density-functional theory or more extensive QM/MM studies, however, supported the model shown in Fig. 6C-a [107, 114]. Since then, X-ray crystallographic studies of urate-bound demolybdo-XOR (Fig. 6C) and the urate-bound reduced Mo form (Fig. 6D) have been reported [105]. The occupancy was only around 60 %, and the broad electron density suggests a mixture of *sp* ^3^ and *sp* ^2^ modes; the latter might be a reaction intermediate during the reaction of xanthine hydroxylation, since it is very unstable and can be obtained at high concentration of urate. Although Glu802 was proposed to promote tautomerization of xanthine in the alternate binding mode, the water molecule located at 3N and 9N of xanthine (Fig. 6C, D), should serve to assist release of the urate product [13, 114]. Figure 6B shows a hypoxanthine-bound desulfo form of XOR determined in our laboratory (to be published elsewhere) that is quite different from the structure proposed by Cao et al. [106]. We have also examined the structures of a number of xanthine-bound desulfo-form XORs, but we have not obtained a clear and consistent electron density even at high resolution. We think the reason for this may be that the active-site cavity of the desulfo form can take a variety of structures for binding various substrates, indicating that X-ray structures of the desulfo form are not appropriate for discussing catalytically relevant binding modes of substrates. Subsequently, Hille et al. [15, 106, 109] presented several critiques of the urate-binding mode [105] and they provided additional data derived from site-directed mutagenesis of the *R*. *capsulatus* enzyme followed by kinetic studies after submission of this manuscript [115]. However, those kinetic data obtained with the mutant enzyme can also be explained in terms of the alternative binding mode described by Metz and Thiel [108] and the bacterial XDH exhibits different molecular dynamics around the active-site cavity from the bovine enzyme [97] that may influence the release kinetics. The focus of discussion has changed from the initial activation energy [112] to the release kinetics, and the issue of the substrate-binding mode still requires clarification.

## Metabolic and pharmacological effects of XOR inhibition

**Fig. 7:**
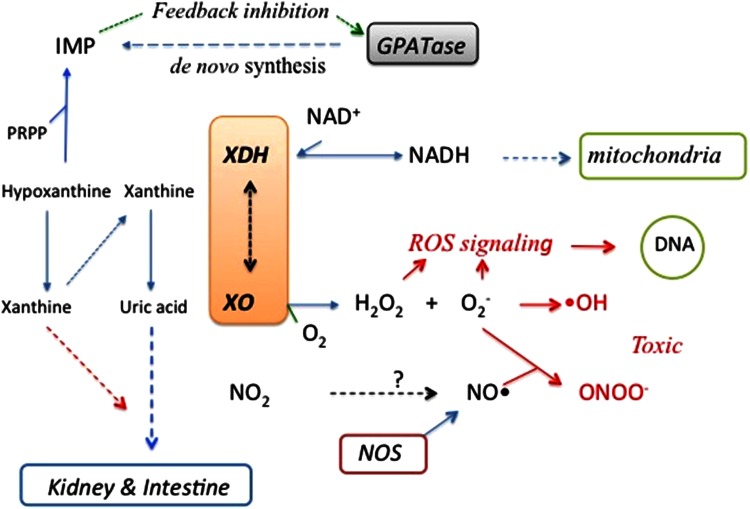
Schematic presentation of possible metabolic and pathological roles of XOR. *ROS* reactive oxygen species, *NOS* nitric oxide synthetase. ·OH can be produced under special conditions

Possible metabolic and pathological roles of XOR are summarized schematically in Fig. 7. Metabolic effects of inhibition can generally be examined in experimental animals or humans. However, higher animals other than primates convert uric acid to more soluble allantoin (Fig. 1), catalyzed by peroxisomal uricase; and inhibitors of uricase, such as oxonic acid, are used for experiments in model animals, such as mouse or rat. In these animals, xanthine is accumulated and secreted into urine via kidney. However, hypoxanthine is less markedly accumulated, being mostly converted to IMP via hypoxanthine guanine phosphoribosyl transferase in the salvage pathway with consumption of phosphoribosyl pyrophosphate (PRPP) (Fig. 1). Accumulated ribonucleotides (AMP and GMP) inhibit the enzyme catalyzing the initial step of purine de novo synthesis, glutamine phosphoribosyl amidotransferase (GPATase) (Fig. 7), in an allosteric manner [116]. The combined effect of this feedback allosteric inhibition and consumption of PRPP might substantially reduce the size of the purine substrate pool, and this might be an effective strategy to treat hyperuricemia. On the other hand, most of the accumulated xanthine is secreted into urine. However, these animals do not seem to have tolerance for severe xanthinuria, which causes kidney damage due to xanthine stone formation [117–120] and often leads to bladder cancer [121]. Urolithiasis is sometimes accompanied with xanthinuria due to xanthine deposition, and this may occasionally lead to acute renal failure [122–127]. On the other hand, human XOR-deficient patients, possible models for inhibition of XOR, do not have such severe symptoms except in the case of type III XOR deficiency, i.e., triple deficiency of XOR, aldehyde oxidase and sulfite oxidase, due to a defect in the synthesis of Moco. Symptoms of Moco deficiency include severe neurological disorder, lens dislocation and dysmorphism, and the outcome is poor [128]. Type I xanthinuria is due to a genetic defect of XOR protein, and Type II, combined deficiency of XOR and AO, is due to a genetic defect of the enzyme catalyzing sulfide incorporation into Moco [129]. Thus, therapeutic usage of XOR inhibitors is not expected to have severe side effects if the inhibitor has no other effect than inhibition of XOR itself. However, it is still possible that patients with such genetic dysfunctions may have compensatory system(s), since systematic genetic analysis has not been carried out in xanthinuria patients [130]. It was reported that XOR can convert nitrite to nitric oxide (NO) [131–135], a vasodilator, and XOR-produced NO may have a protective role in myocardial ischemia [136, 137]. Thus, potent XOR inhibitors may be contra-indicated for patients having cardio-vascular disease. However, it should be noted that NO production by XO is very low even under anaerobic conditions, i.e., roughly ~1 % of urate formation activity from xanthine [50, 138]. Maia and Moura [135] reported NO formation by AO with aldehyde substrates. It is not surprising from a chemical point of view that the water-exchangeable hydroxyl group at OH–Mo(IV) can be replaced by NO_2_ to produce NO under strict anaerobic conditions, since various compounds, such as urate, oxipurinol and FYX-051 derivatives, can behave similarly. Nevertheless, although formation of NO is chemically feasible under strict anaerobic conditions, it is not yet clear whether these reactions have any physiological significance. Clinical studies of allopurinol, febuxostat and topiroxostat are due to start in EU countries [139].

As XOR has the potential to produce reactive oxygen species (ROS), inhibitors of XOR might be effective in treating XOR-associated diseases [140]. XOR exists as the XDH form, which does not produce ROS, under normal conditions in vivo, where NAD^+^ exists dominantly in cytosol, because it can be isolated as the XDH form by very careful extraction procedures. Thus, production of ROS requires conversion of XOR to XO under conditions where disulfide formation or proteolysis can occur, and then XO must be secreted into the blood. The resulting XO produces H_2_O_2_, O_2_^−^ and highly active hydroxyl radical. Further, O_2_^−^ scavenges NO produced by nitric oxide synthetase, reacting with NO to give toxic peroxynitrite (Fig. 7). On the other hand, uric acid is known to be a good anti-oxidant [141, 142]. The potential clinical utility of XOR inhibitors still remains to be established, although there are number of reports regarding this issue [140]. Nevertheless, XOR inhibitors should be useful tools to examine the pathophysiological significance of ROS generation by XOR.

Finally, we should comment on the possible interaction of XOR inhibitors with aldehyde oxidases (AOXs), which are also members of the molybdo-flavoenzyme family and have various physiological roles [143, 144]. All AOXs are very similar to XOR in molecular weight, cofactor composition and three-dimensional structure [145], but key residues for catalytic activity are not completely conserved in the active-site cavity, except for the glutamate residue located near the Mo atom that is important for catalytic activity. As already noted, the active-site residues are important not only for substrate specificity [40, 146], but also for binding affinity of XOR inhibitors. Further, even highly potent XOR inhibitors cannot effectively inhibit bacterial XDH, although only minor amino acid differences are observed in the Moco domain structures that influence the molecular dynamics around the active-site cavity [97]. Thus, we think it is unlikely that highly potent XOR inhibitors would interact with AOXs. Conversely, highly potent AOX inhibitors may not interact with XOR. Nevertheless, this issue deserves further study, although as described already it is known that patients with combined XO and AOX (type II xanthinuria) deficiency due to loss of Moco sulfurase [129] do not exhibit any severe symptoms [130, 147].
